# An Investment Case to Prevent the Reintroduction of Malaria in Sri Lanka

**DOI:** 10.4269/ajtmh.16-0209

**Published:** 2017-03-08

**Authors:** Rima Shretta, Ranju Baral, Anton L. V. Avanceña, Katie Fox, Asoka Premasiri Dannoruwa, Ravindra Jayanetti, Arumainayagam Jeyakumaran, Rasike Hasantha, Lalanthika Peris, Risintha Premaratne

**Affiliations:** 1 Global Health Group, University of California, San Francisco, California.; 2 Swiss Tropical and Public Health Institute, Basel, Switzerland.; 3 University of Basel, Basel, Switzerland.; 4 Anti Malaria Campaign, Ministry of Health, Nutrition and Indigenous Medicine, Narahenpita, Colombo, Sri Lanka.

## Abstract

Sri Lanka has made remarkable gains in reducing the burden of malaria, recording no locally transmitted malaria cases since November 2012 and zero deaths since 2007. The country was recently certified as malaria free by World Health Organization in September 2016. Sri Lanka, however, continues to face a risk of resurgence due to persistent receptivity and vulnerability to malaria transmission. Maintaining the gains will require continued financing to the malaria program to maintain the activities aimed at preventing reintroduction. This article presents an investment case for malaria in Sri Lanka by estimating the costs and benefits of sustaining investments to prevent the reintroduction of the disease. An ingredient-based approach was used to estimate the cost of the existing program. The cost of potential resurgence was estimated using a hypothetical scenario in which resurgence assumed to occur, if all prevention of reintroduction activities were halted. These estimates were used to compute a benefit–cost ratio and a return on investment. The total economic cost of the malaria program in 2014 was estimated at U.S. dollars (USD) 0.57 per capita per year with a financial cost of USD0.37 per capita. The cost of potential malaria resurgence was, however, much higher estimated at 13 times the cost of maintaining existing activities or 21 times based on financial costs alone. This evidence suggests a substantial return on investment providing a compelling argument for advocacy for continued prioritization of funding for the prevention of reintroduction of malaria in Sri Lanka.

## Introduction

Sri Lanka has made extraordinary gains in reducing the burden of malaria in the last decade. Between 2000 and 2011, the number of malaria cases declined by more than 99%.^1^,^2^ With zero locally transmitted malaria cases recorded since November 2012 and no indigenous deaths since 2007, Sri Lanka received the World Health Organization (WHO) certification of elimination in September 2016, an official recognition of its malaria-free status.^1^,^3^,^4^ This period of progress coincided with increased political and financial commitment from the government and external donors, particularly the Global Fund to Fight AIDS, Tuberculosis and Malaria (Global Fund).

As Sri Lanka's national malaria program, the Anti Malaria Campaign (AMC), shifts its programmatic focus toward prevention of reintroduction (POR), it faces a new set of strategic and financial challenges.^5^ Funding for malaria from the Global Fund is declining and being prioritized for high-burden, low-income countries.^6^ At the same time, there is waning political interest and a rising disinterest toward malaria among health workers within the country as the disease is no longer considered a major public health threat and other health issues such as dengue fever and noncommunicable diseases have become more pressing national health priorities.^5^

Abruptly shifting focus away from the malaria program at this critical juncture is a conceivable risk to malaria resurgence in Sri Lanka. Scaling down of malaria efforts due to funding withdrawal in Sri Lanka in the 1960s is arguably the most cited resurgence story in history.^7^ In 1963, malaria elimination was on the horizon with only 17 cases recorded in public facilities, of which only six were autochthonous (locally transmitted).^2^,^8^ Following this success, there was a severe cutback in political and financial support for malaria control, leading to the withdrawal of malaria control measures, weakened surveillance and programmatic support, and growing insecticide resistance. Rapid resurgence of malaria soon followed with confirmed malaria cases rising to more than half a million in 1969.^8^ Between 1970 and 1999, malaria control interventions were resumed; however, frequent epidemics continued to occur during the 1980s and early 1990s.

The country continues to face a significant risk of resurgence especially in areas of high receptivity and vulnerability. Increased levels of tourism, migration, poor infrastructure in some areas, and the presence of vectors contribute to vulnerability to autochthonous transmission triggered by imported malaria.^9^,^10^ In 2013, 95 imported cases of malaria were reported throughout the year. Sixty percent of the imported cases occurred among Sri Lankans returning from travel overseas, most being diagnosed and reported by public sector hospitals in the Western Province, an area not traditionally endemic for malaria.

To counter these challenges, Sri Lanka embarked on a new national strategic plan (NSP) for the elimination and POR of malaria for 2014–2018.^5^ The key focus of this strategy was to reorient and focus the program to strengthen surveillance systems for malaria, to facilitate rapid detection and response to emergent cases, and to eliminate parasite reservoirs and transmission foci. To implement this strategy, the AMC needs continued resources particularly in the short- to medium-term until the intrinsic transmission potential is sufficiently altered to make elimination stable.

The purpose of this study was to develop an investment case for malaria POR in Sri Lanka. In addition, it reviews the funding landscape for malaria in the country and identifies anticipated gaps in the near future. The findings will provide the AMC with an estimate of the resources required to prevent the reintroduction of malaria, as well as robust evidence to advocate for sustained funding from both domestic and external sources.

## Methods

### Study design.

This study used a cost–benefit approach in which the cost of current malaria program activities was computed against the economic benefits of maintaining the program. A comprehensive literature review was initially conducted to gain an understanding of the current and historical structure, activities, and financing of the malaria program.

A micro-costing approach was used to obtain data on the costs of POR. A detailed cost analysis was conducted for ongoing program activities from expenditure and financial records, historical record reviews as well as extraction from existing reports and key informant interviews. Available information was obtained from existing reports and grey and published literature, including AMC records at the national and regional levels.

All fixed and recurrent costs incurred by the health system for malaria activities including resources received as donations and other in-kind or indirect expenditures were captured. Costs were categorized by source of funding, type of cost input, and by activity or intervention. Benefits were measured as the averted costs of resurgence were estimated under a hypothetical scenario of resurgence, which was constructed based on historical data and expert opinion in the country. Under this counterfactual scenario, it was assumed that all POR activities would be halted in 2014 resulting in an increase in malaria cases between 2015 and 2020 with a peak in 2017, mimicking the magnitude and trend of the malaria epidemic between 1997 and 2002, adjusted for population growth. The cost of resurgence was estimated as the direct and indirect cost incurred by the health system to prevent and treat the increased cases as well as the direct and indirect cost incurred by individual households and the society.

**Figure 1.**
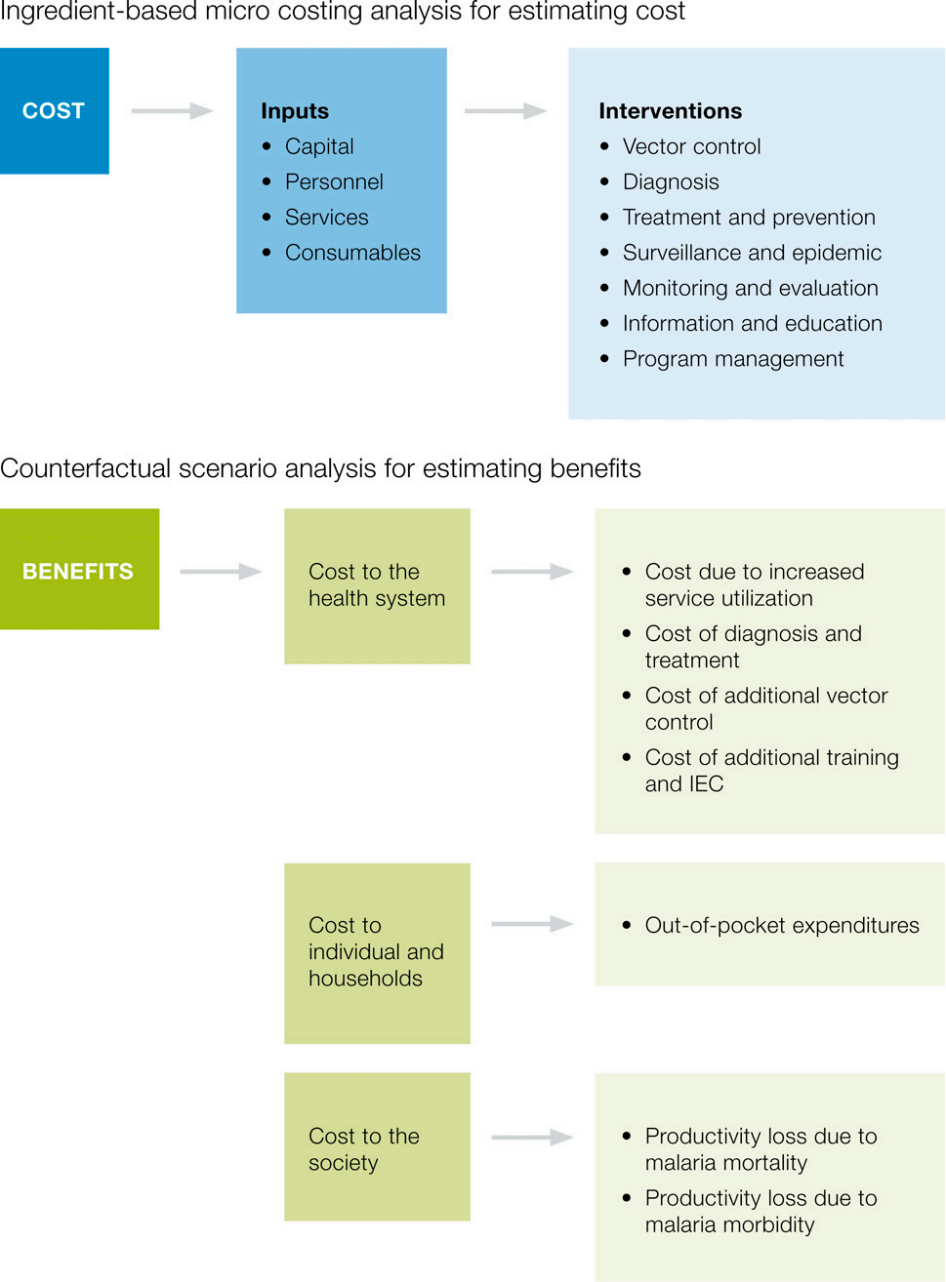
Framework for cost and benefit analysis.

The framework presented in Figure 1

was used to develop the cost–benefit analysis using an ingredient-based micro-costing analysis for estimating cost and a corresponding counterfactual scenario analysis for estimating benefits.

### Study setting and sampling.

Sri Lanka is divided into nine provinces and 25 administrative districts. We purposively sampled five districts in five different provinces to collect data on the cost of the malaria activities for POR: Hambantota (Southern Province), Ampara (Eastern Province), Anuradhapura (North Central Province), Puttalam (North Western Province), and Jaffna (Northern Province). The sampled districts represented regions where recent cases had been identified and included a range of previously endemic regions that used different mixes of interventions. Based on input from the AMC and other in-country experts, these sampled districts were deemed to be representative of the remaining 20 districts with respect to programmatic costs and levels of receptivity and vulnerability to malaria transmission. In addition, cost data were also collected from the AMC at the national level.

### Data collection.

Data collection for this study took place between February and July 2015. Data on the costs of malaria POR activities for 2014 were obtained from interviews and a review of the most recent budget and expenditure records. Staff at the regional malaria offices (RMOs) in each of the sampled districts was interviewed in a semi-structured format. The time spent on each activity was recorded based on self-reporting by the RMOs and other interviewees triangulated with interviews with the AMC director. At the central level, officers at the AMC including the AMC director, director of finance and accounting, surveillance, and monitoring and evaluation unit staff, and the Global Fund project finance manager were interviewed.

Data for the cost of resurgence were retrieved from published and unpublished literature and described in detail under “data analysis” below. Key informant interviews with AMC staff were also conducted to obtain consensus on the assumptions used and to fill any outstanding data gaps. The data on financing for malaria were extracted from existing reports and grey and published literature including, but not limited to, Internet-based searches and AMC records at the national and regional levels.

This study was approved by the institutional review boards of the University of California, San Francisco Committee on Human Research (Study no. 14-14546, Reference no. 093635) and the Ethics Review Committee of the Faculty of Medicine, University of Kelaniya, Sri Lanka (Reference no. P/209/10/2014). Verbal informed consent procedures were conducted before each interview.

### Data analysis.

#### Estimating cost of POR.

Primary data on costs collected from each sample district and the AMC were aggregated based on three dimensions—funding source, activity or intervention, and input type—to identify the cost drivers for malaria POR activities. All costs were expressed in 2013 U.S. dollars (USD), using a mid-year exchange rate of 131.5 Sri Lankan rupees per USD.

##### Cost by source.

The two main sources of funding for malaria activities in Sri Lanka were 1) domestic funding, in the form of direct government allocations from the national health budget to the AMC and to the provinces, and 2) external funding, primarily from the Global Fund provided to the government for malaria activities. Government resources were disbursed to provinces and districts for all integrated health activities including malaria prevention and control separately from the resources provided to the AMC specifically for malaria activities. The explicit source of funding for malaria activities for each line item was identified to the extent possible.

##### Cost by input.

Costs were categorized by four major inputs of production: capital, personnel, consumables, and services. Capital costs included vehicles, buildings and office space, furniture, computers, and other durable supplies. Personnel costs included salaries, allowances, and any other compensation to staff involved in malaria activities. Consumable costs included office and laboratory supplies, medicines, insecticides, and other products. Service costs included utilities, transport (domestic and international), training, maintenance, and security.

Capital goods were annualized based on their useful life years and a standard discount rate of 3%. Maintenance costs for equipment, vehicles, or buildings were calculated using actual information on the expenditure of maintaining these resources. No replacement costs were used for capital resources when their current value had already depreciated to zero, assuming that replacement would not occur in the near future. For all inputs shared across multiple programs, only the cost attributed to malaria activities was included based on the percentage of time spent on malaria-specific activities. Shared resources such as staff time spent on each activity were self-reported and determined through interviews and triangulated using multiple sources.

##### Cost by activity or intervention.

All costs were divided across seven different activity groups for malaria: vector control (VC); diagnosis (D); treatment and prophylaxis (TP); surveillance and epidemic management (SEM); monitoring and evaluation (ME); information, education, and communication (IEC); and program management (PM). Although the implementation of most of these activities was integrated, the activity groups were created to facilitate analysis for the purpose of this study. Resources were apportioned across the activities based on self-reporting during interviews. Table 1 details the inputs for each of these interventions.

##### Estimating cost of POR at the national level.

To obtain national-level estimates of cost of POR, data from the five sampled districts were extrapolated to the entire country by matching each non-sampled district to a representative sampled district. District matching was based on the size of the malaria program and the mix of activities implemented by the sampled and non-sampled districts. The number of staff and the size of the district measured by area in square kilometers were used as proxies for the size of the malaria program for the purpose of matching for cost extrapolation. Districts in the Western Province (i.e., Colombo, Gampaha, and Kalutara) were not matched in the same way because the AMC serves as the RMO for this region and their costs were already incorporated into AMC costs. To estimate the national cost of POR, the total cost incurred by each sample district in 2014 was divided by its respective population to get the average cost per capita. The population of each non-sampled district was multiplied by the average cost per capita from the corresponding matched sample district. Costs across all districts were then summed together with the central level costs from the AMC to estimate the total cost of POR for the country for 2014. The AMC anticipated that the activities and, therefore, the cost of continuing POR over the next 3–5 years is likely to be similar to the cost of the program in 2014. The NSP (2014–2018) prioritizes strengthening of the existing interventions for malaria, particularly surveillance and response for the early detection of cases and their effective treatment, maintaining skills for diagnosis and treatment, strengthening preparedness for epidemic and outbreak response, and entomological surveillance through integrated vector management. The cost data estimated for 2014 were thus projected linearly to obtain cost estimates for 2015–2020, assuming a steady economic growth rate.

### Estimating cost of resurgence.

The benefit of sustained investments in malaria and hence the corresponding cost saving from POR activities was obtained by estimating the cost of potential malaria resurgence. A hypothetical resurgence scenario was constructed based on the assumption that all POR activities would have been halted in 2014 resulting in an increase in malaria cases between 2015 and 2020 similar to that observed during the epidemic between 1997 and 2002, after adjusting for population growth. In this scenario, the peak number of malaria cases was assumed to be 324,371 with 122 deaths with a total epidemic size of 1,241,776 cases. The detailed parameters used to estimate the cost of resurgence and their data sources are listed in Table 2. As shown in Figure 1, the costs of resurgence were categorized based on three broad dimensions: 1) cost to the health system, 2) cost to the individual households, and 3) cost to the society.

#### Cost to the health system.

##### Cost due to increased health service utilization.

The potential cost of malaria resurgence to the health system was calculated separately for uncomplicated malaria (UM) and severe malaria (SM). Of the UM cases, *Plasmodium vivax* cases were presumed to be treated with primaquine for 14 days and chloroquine for 3 days according to the national treatment guidelines, and *Plasmodium falciparum* cases with artemether–lumefantrine as inpatients. Table 3 outlines the malaria treatment guidelines in Sri Lanka.

The unit costs of malaria treatment were multiplied by the number of potential cases to estimate the total cost of treatment to the health system. Actual health system costs for both inpatient and outpatient treatment of malaria were not available as malaria services are integrated with general health services. Therefore, secondary data from a separate micro-costing database from a teaching hospital in Kurunegala, Sri Lanka, were used to approximate service delivery costs, which included the average cost of outpatient care (including consultation and diagnostic tests) and the average cost of hospital admission for all patients regardless of original complaint or final diagnosis (Kurunegala Teaching Hospital, personal communication). The cost of inpatient care thus includes the length of a hospital stay multiplied by the average cost of a hospital bed per day. The cost of an average course of antimalarials as reported by the AMC was added to this to obtain the total cost of malaria treatment (AMC, personal communication). Supply chain costs were estimated as 25% of the acquisition cost of the product and added to the unit cost of the medicine.^11^

##### Cost of vector control.

The cost of indoor residual spraying (IRS) and distribution of long-lasting insecticidal nets (LLINs) were used to estimate the cost of vector control under the resurgence scenario. Under this scenario, we assumed that the country would resume IRS at a coverage rate of 4% of the total population, similar to the coverage rate during the 1999 resurgence (AMC, personal communication).^21^

In addition, LLIN coverage of 1 net per 1.8 people was assumed, based on WHO recommendations for the population at risk.^15^ The total population at risk was identified in collaboration with the AMC based on the receptivity and vulnerability for malaria transmission in the country. Costs for procurement, distribution, and delivery of LLINs and IRS were obtained from WHO Global Malaria Program and added to the cost of vector control as these costs were not available in-country (Patouillard, E., personal communication).

##### Cost of increased diagnosis of fever cases for malaria.

Under the resurgence scenario, it was assumed that more fever cases would be tested for malaria, leading to increased spending on rapid diagnostic tests (RDTs) and microscopy. Using the slide positivity rates from 1999 of 16.72% and the expected number of positive malaria cases in 2015, we estimated the total number of potential non-malaria cases assuming that 83.28% of the cases would be non-malarial fevers. The excess cost of diagnosing non-malaria fever cases was obtained by multiplying the number of potential non-malarial fevers by the average cost of diagnosis (average of RDT and microscopy) plus the cost of administering the test (AMC, personal communication).^22^

##### Cost of training and IEC.

In the event of malaria resurgence, it was assumed that there will be additional training for providers of all cadres, as well as additional IEC-related activities directed at the community.

#### Cost to the individual household.

##### Out-of-pocket (OOP) expenditures incurred due to malaria.

All malaria cases are treated in the public sector free of charge. They do not incur any user fees and there are no social health insurance schemes covering malaria. OOP expenditures due to malaria include both direct and indirect cost incurred by the household for preventing or seeking care for malaria. These included transport costs as well as expenditures on other products for prevention, such as LLINs, mosquito coils, and repellents. These expenditures were extrapolated from secondary data from a study done in Sri Lanka in 1994 and inflated to reflect current costs (N. Attanayake, unpublished data).^17^

#### Cost to society.

##### Cost due to loss of life to malaria.

The full income approach (see equation below) was used to estimate the potential social value of life lost due to malaria mortality as proposed by the Lancet Commission on Investing in Health.^23^ This approach combines growth in national income with the value of additional life years (VLYs) due to malaria, which accounts for an individual's willingness to trade off income, pleasure, or convenience for an increase in life expectancy.

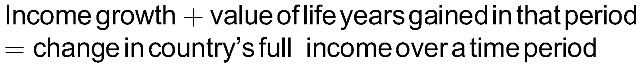



The (potential) number of adult deaths due to malaria in the resurgence scenario was multiplied by the remaining life years at death and the VLYs. The number of excess deaths among adults (persons age 15 years and above) in the hypothetical scenario were projected based on deaths between 1997 and 2002. The average life expectancy at age 40 years (separately for male and female) obtained from World Bank data was used as a proxy for the remaining life years at death due to malaria.^11^ The Lancet Commission estimates the VLY average across low- and middle-income countries to be 2.3 times the income per capita at a 3% discount rate.^23^,^24^ Sri Lanka's gross domestic product (GDP) per capita at 7.4% for 2015 was obtained from the World Bank database.^14^

##### Cost due to loss of productivity to malaria morbidity.

The reduced productivity or lost earnings due to malaria morbidity in adults was estimated by multiplying the potential malaria cases among the adult population, average days lost to one malaria episode (estimated at 9.3 days from previous research), and the average income (GDP) per capita per day obtained from World Bank data.^14^,^16^

### Estimating return on investment.

The return on investment (ROI) to the health system was calculated as the difference in total cost of POR and total cost of potential resurgence, also known as the net gain, divided by the total cost of POR. The cost of POR was computed from an input perspective using data from the costing portion of this study, whereas the cost of resurgence was computed from an output perspective, where the output costs were multiplied by the potential number of cases under resurgence.

### Uncertainty analysis.

As with any cost and benefit estimation, our estimates relied on various assumptions about the input parameters, such as discount rates. To test the sensitivity of the cost of POR to discounting, the discount rate used for capital goods was varied between 1% and 7%.

Another key underlying assumption in this analysis is that withdrawal of all malaria interventions will result in resurgence. The risk of resurgence in the future primarily hinges on two key parameters: the probability of resurgence and the severity of resurgence. In our construction, we assumed that future resurgence will be as severe as that experienced during the most recent epidemic between 1997 and 2002 (with a peak in year 1999). We also assumed that the resurgence would follow a similar distribution pattern and that a resurgence of this severity would occur with a 100% probability.

To assess the robustness of our estimates with regard to the uncertain risk of resurgence, we conducted a sensitivity analysis by generating several alternative scenarios of resurgence with varying assumptions of severity and probability based on historical data. Following the application in the insurance industry and recent literature on pandemic influenza risk, we used the notion of “exceedance probability” to test probability of a resurgence with a certain threshold severity. Using historical data on malaria incidence, the maximum annual growth rate and the maximum total growth rate (between trough years) were used to vary the severity levels. Additional probabilities for the risk of resurgence were based on available historical data in the literature. Cohen and others (2012) noted that 75 malaria resurgence events occurred over 70 years in 61 different countries, which translates to a 2% probability of resurgence. We used this as a lower bound estimate to analyze the sensitivity of the ROI to varying probabilities of resurgence between 2% and 100%.

**Figure 2.**
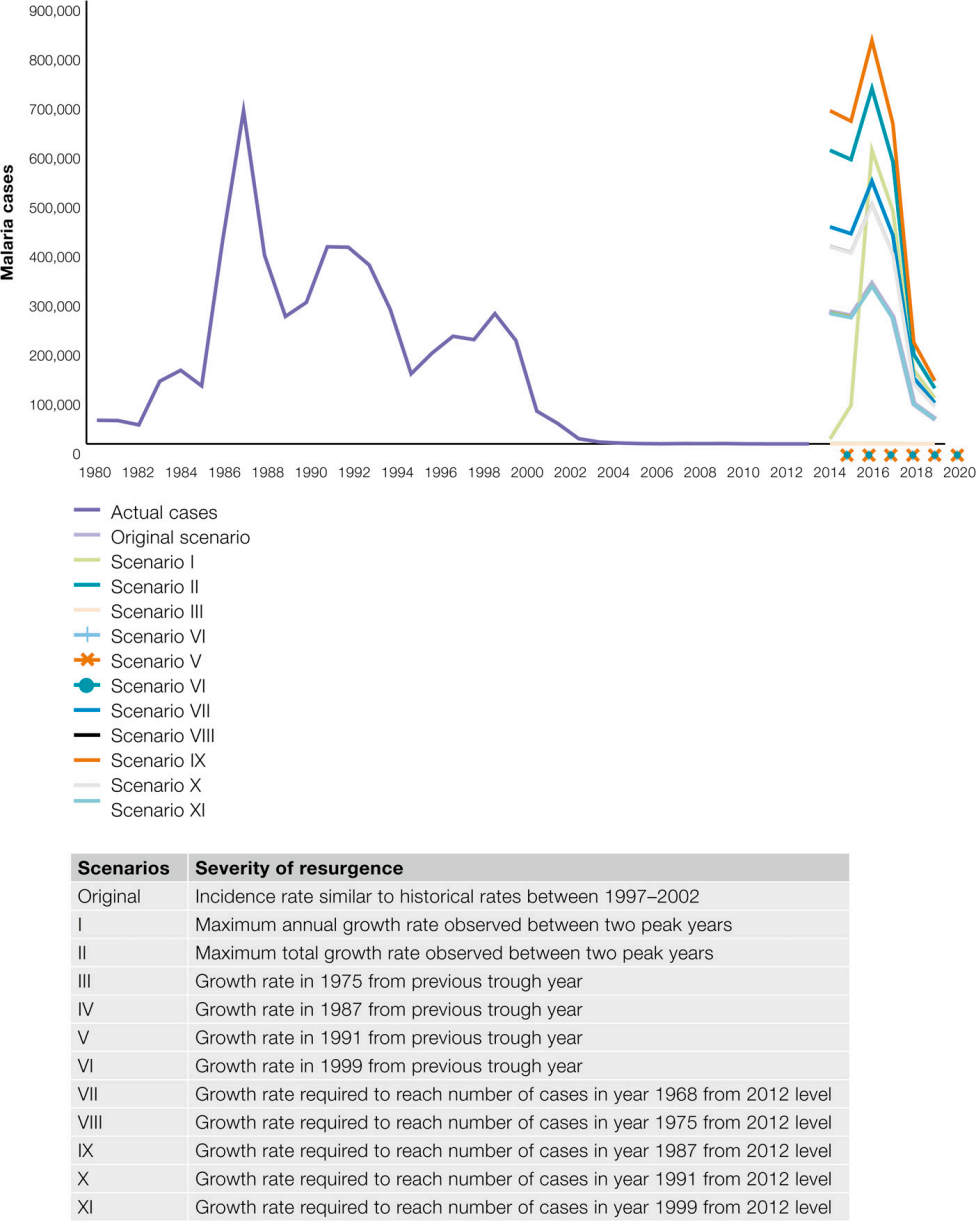
Framework for uncertainty analysis.

Table 4 includes the various parameters that were varied and scenarios that were generated to assess the uncertainty of the cost and ROI estimates. Figure 2

illustrates the scenarios that were as translated into incidence projections.

### Financial costs of malaria.

The financial costs of malaria POR were obtained from the estimates of economic costs without accounting for capital costs or the cost of the general health system or personnel that are financed through integrated national and provincial health budgets not specific to malaria.

## Results

### Cost of POR.

The total economic cost of the malaria program in Sri Lanka for 2015 was estimated to be USD11.85 million (Table 5). Fifty eight percent of the total cost was incurred by the AMC, whereas the remaining 42% was incurred by the provincial level. Cost estimates varied widely across the districts from less than USD30,000 to about USD0.5 million per year with a median cost of USD197,252. The average economic cost per capita for POR was estimated to be USD0.57 and the corresponding financial cost was USD0.37 for 2015.

About 80% of the total cost was funded domestically, of which 8% was from provincial funds and 72% was from national government. The remaining 20% was financed by the Global Fund. Funding for the AMC was primarily domestic (82%) and the remaining 18% from the Global Fund. Across the districts, the source of funding varied largely with an average of 70% domestic (of which 9% was national and 62% was provincial) and 29% from donors.

**Figure 3.**
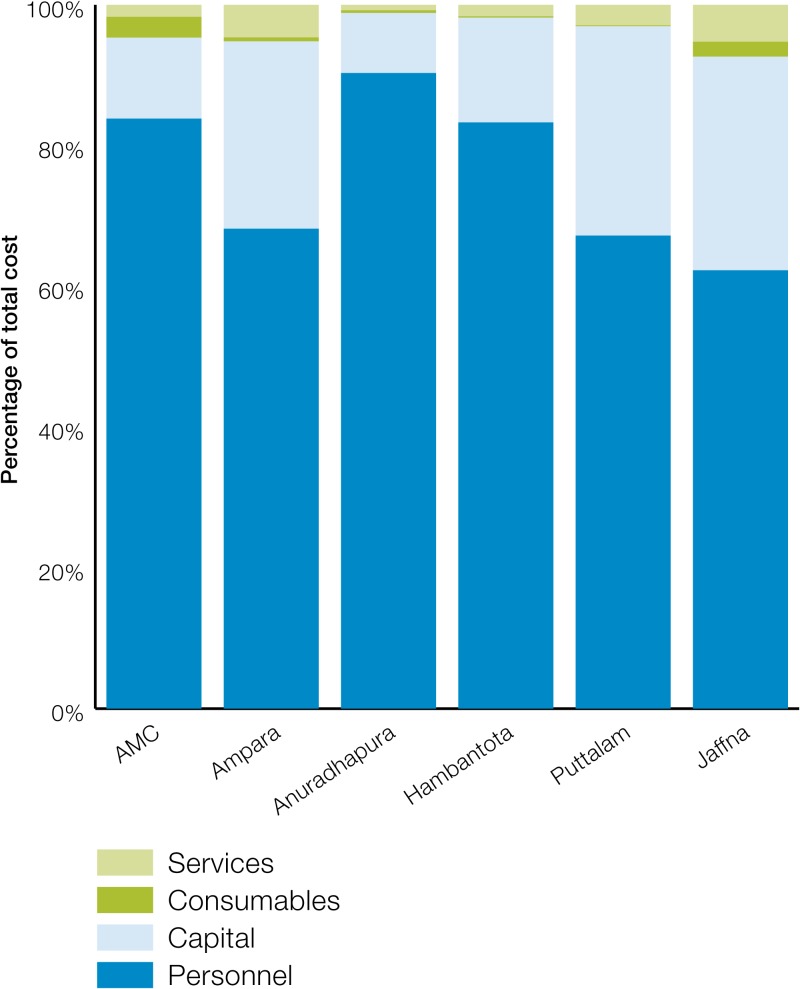
Distribution of input cost across sample districts.

Among the inputs, human resources constituted the largest share at about 83% of the total cost, followed by capital costs at about 13%. Consumables and services together constituted about 5% of total cost of malaria POR. There was considerable heterogeneity in the mix of inputs across the district and the national levels (Figure 3

). In all instances, however, human resources were the main cost driver at 62–90% of total cost among the districts. The share of capital cost was, on average, 22% (range: 8–30%). Consumables constituted < 1% of the total cost (range: < 1–3%), and services constituted approximately 3% of the total cost (range: < 1–5%).

**Figure 4.**
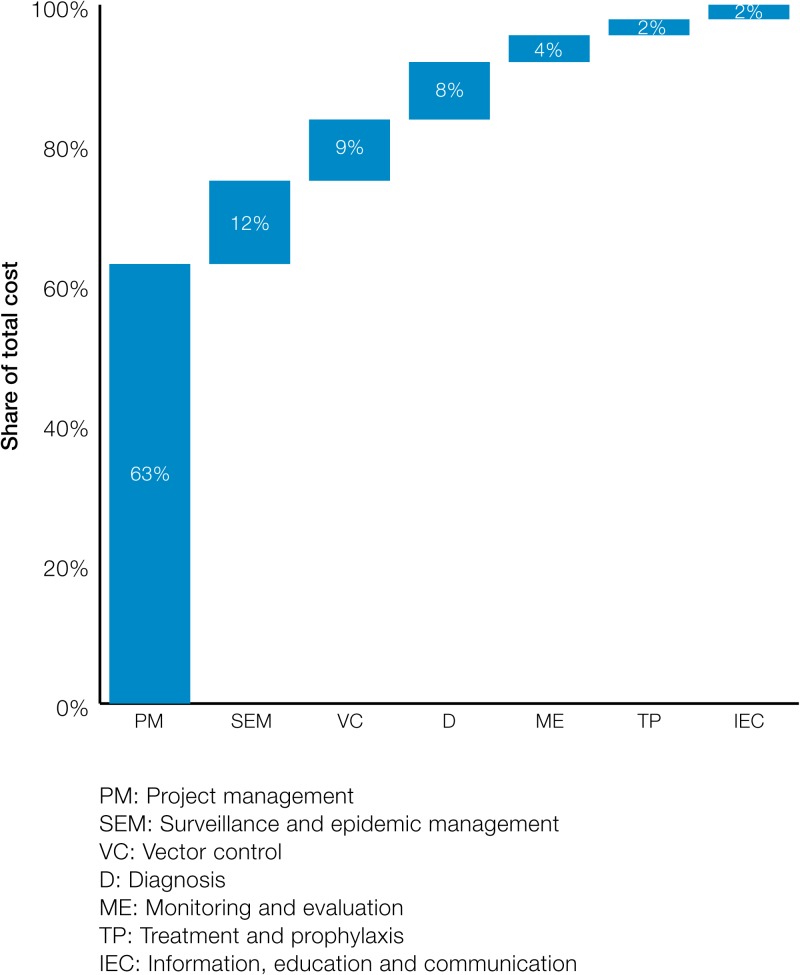
Distribution of total cost of POR across interventions.

Among the activities, the major cost drivers at all levels were project management and surveillance and epidemic management, followed by VC and D. Figure 4

illustrates the distribution of total costs across all activities in Sri Lanka. At a national level, PM consisted of about 63% of the total cost, followed by SEM at about 12%, and VC at 9%.

**Figure 5.**
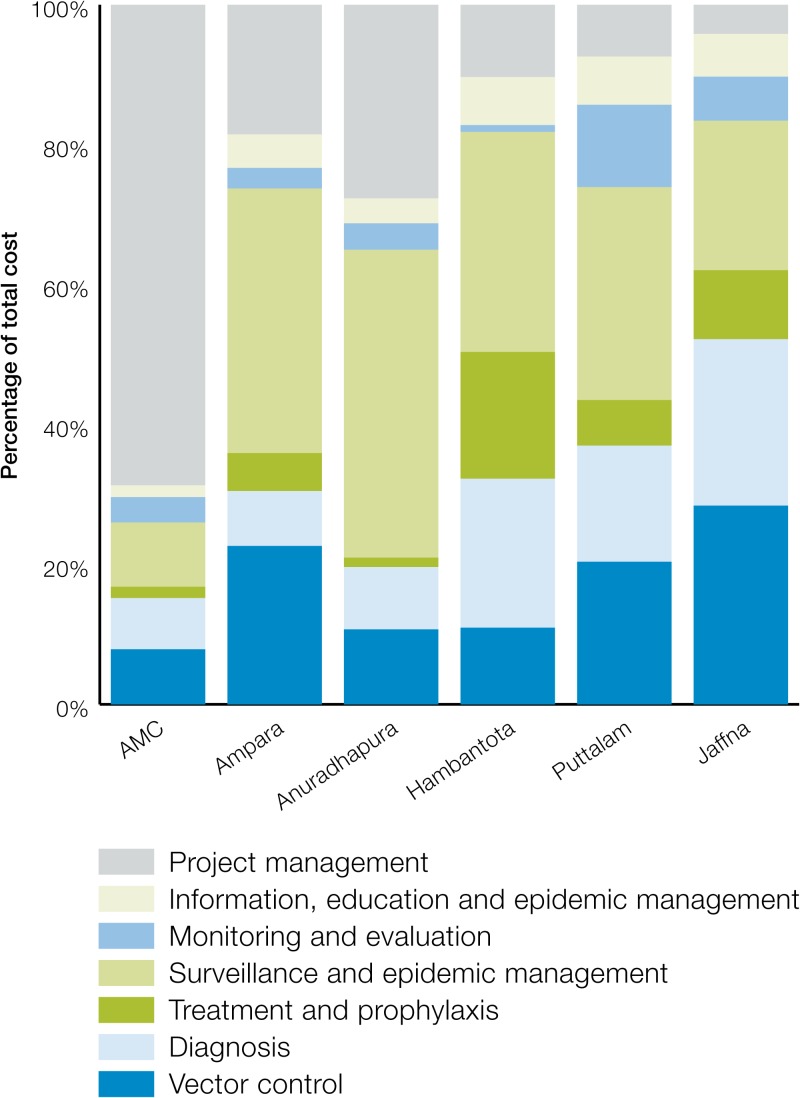
Distribution of cost of POR by intervention across districts.

The cost of activities also varied widely across districts (Figure 5

). At the district level, SEM constituted an average of 33% of the cost (range: 21–44%). Across the districts, the cost share for VC averaged 19% (range: 11–28%). Similarly, the cost share of D ranged between 8% and 24% with an average of 16%. The cost share of IEC was fairly stable across districts at approximately 5% of the total cost.

**Figure 6.**
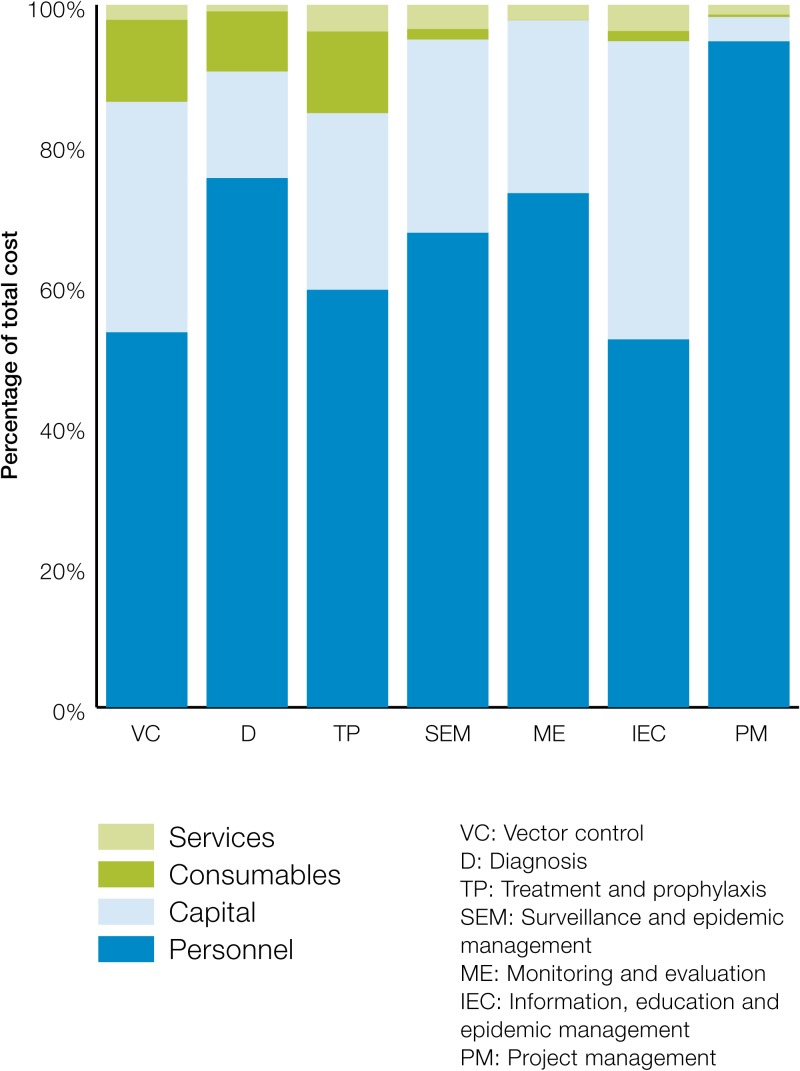
Distribution of input cost across interventions.

Across all activities, human resources constituted the highest share, followed by capital costs. The most human resource-intensive interventions were PM, SEM, and ME. IEC was the most capital-intensive intervention. As expected, TP followed by VC and D constituted relatively higher shares of consumable costs than other interventions. These differences in inputs across interventions are illustrated in Figure 6

.

#### Cost of POR activities over time.

The future cost of POR was extrapolated using the costs for 2014 adjusting for economic growth under the assumption that most of the activities and interventions for POR will remain constant over the next 5 years. The estimated cost to sustain the current level of activities for malaria between 2015 and 2020 was estimated at USD83 million (Table 4).

### Cost of resurgence.

Figure 7

**Figure 7.**
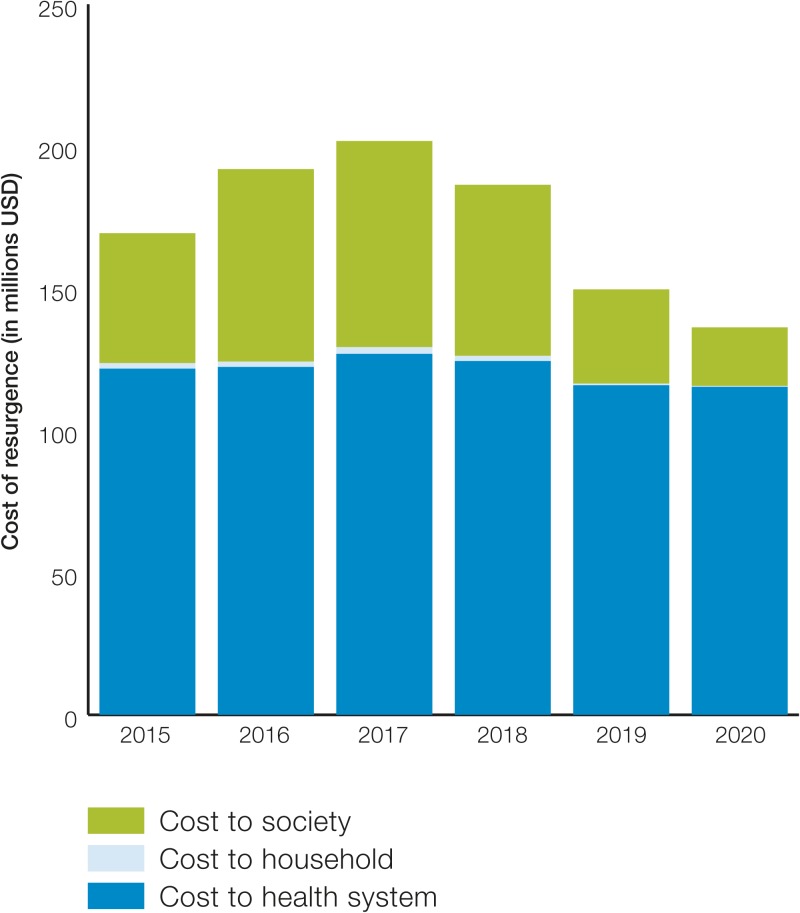
Cost of resurgence of malaria in Sri Lanka.

illustrates this cost of resurgence between 2015 and 2020 broken down by the cost to household, the health system, and society. The total cost of resurgence was estimated at approximately USD169 million. Within this cost, the direct cost to the health system was USD121 million, the cost to households was USD1.95 million, and the cost to society totaled USD45.66 million (Table 6). The cost of resurgence was estimated to be the highest for year 2017 when the incident cases peak and started declining following the trajectory of malaria incidence. The majority of the cost of resurgence is incurred by the health system, followed by the cost to society. As the majority of malaria interventions are publicly funded, out-of-pocket expenditures or household did not constitute a large portion of the cost.

### Return on investment.

The total cost of malaria POR in Sri Lanka for 2015 was estimated to be USD11.86 million, whereas the total cost of resurgence for the corresponding year was USD168.96 million yielding a ROI of more than 13 to 1. When considering the financial costs only (without capital cost and non-malaria-specific cost incurred by the integrated health system), the ROI was estimated at more than 21 to 1.

Similarly, when considering only the financial cost to the health system (without individual or societal costs), the cost of resurgence is estimated to be about 10 times the cost of maintaining the activities yielding a ROI of 10 to 1.

### Uncertainty analysis.

An uncertainty analysis was carried out using a variety of discount rates to test the robustness of the results. The results did not vary significantly with the discount rates used—the difference in the cost estimate between the highest and the lowest discount rates was less than USD0.2 million in 2015. A discount rate of 3% produced the median cost estimates and was retained for this analysis.

**Figure 8.**
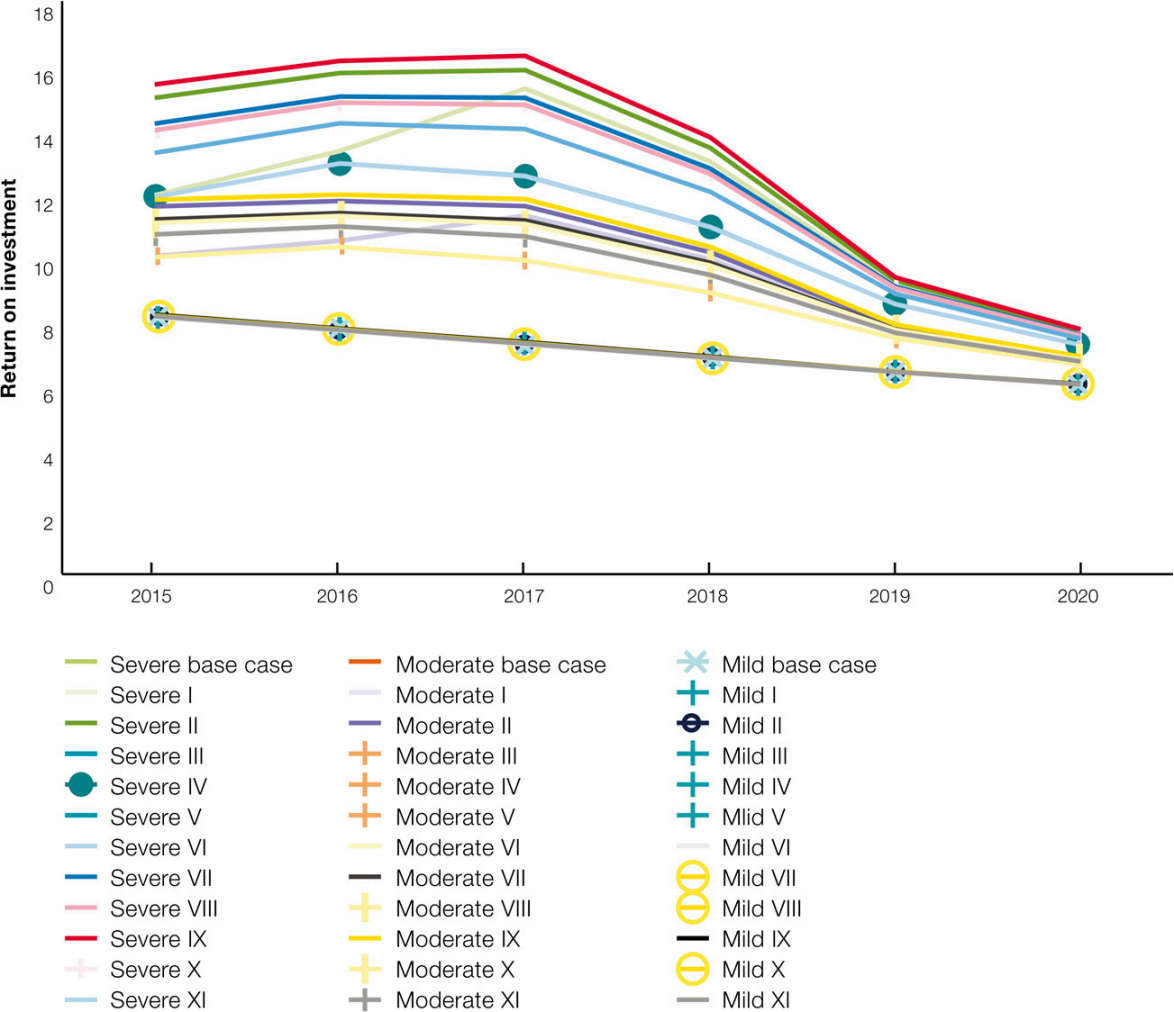
Sensitivity analysis of the estimates of return on investment in malaria using economic costs.

In addition, a sensitivity analysis was performed on the cost of resurgence by varying the risk and probability of a hypothetical resurgence scenario. Figure 8

illustrates the ROI obtained under the various scenarios. Under these resurgence scenarios, the cost of resurgence was estimated at between USD78 and 208 million. The ROI, in turn was found to be between 6 and 16 under various severity and probabilities of resurgence. As expected, the cost of resurgence starts declining as the resurgence is contained after the peak year in 2017, resulting in a subsequent reduction in ROI. Figure 8 illustrates the sensitivity of the ROI using economic cost to varying levels of risk and probability of resurgence. Eleven scenarios of incidence were used in the sensitivity analysis denoting risk at three levels of probability: 100%, 51%, and 2% for a total 33 scenarios (Table 4).

Figure 9

**Figure 9.**
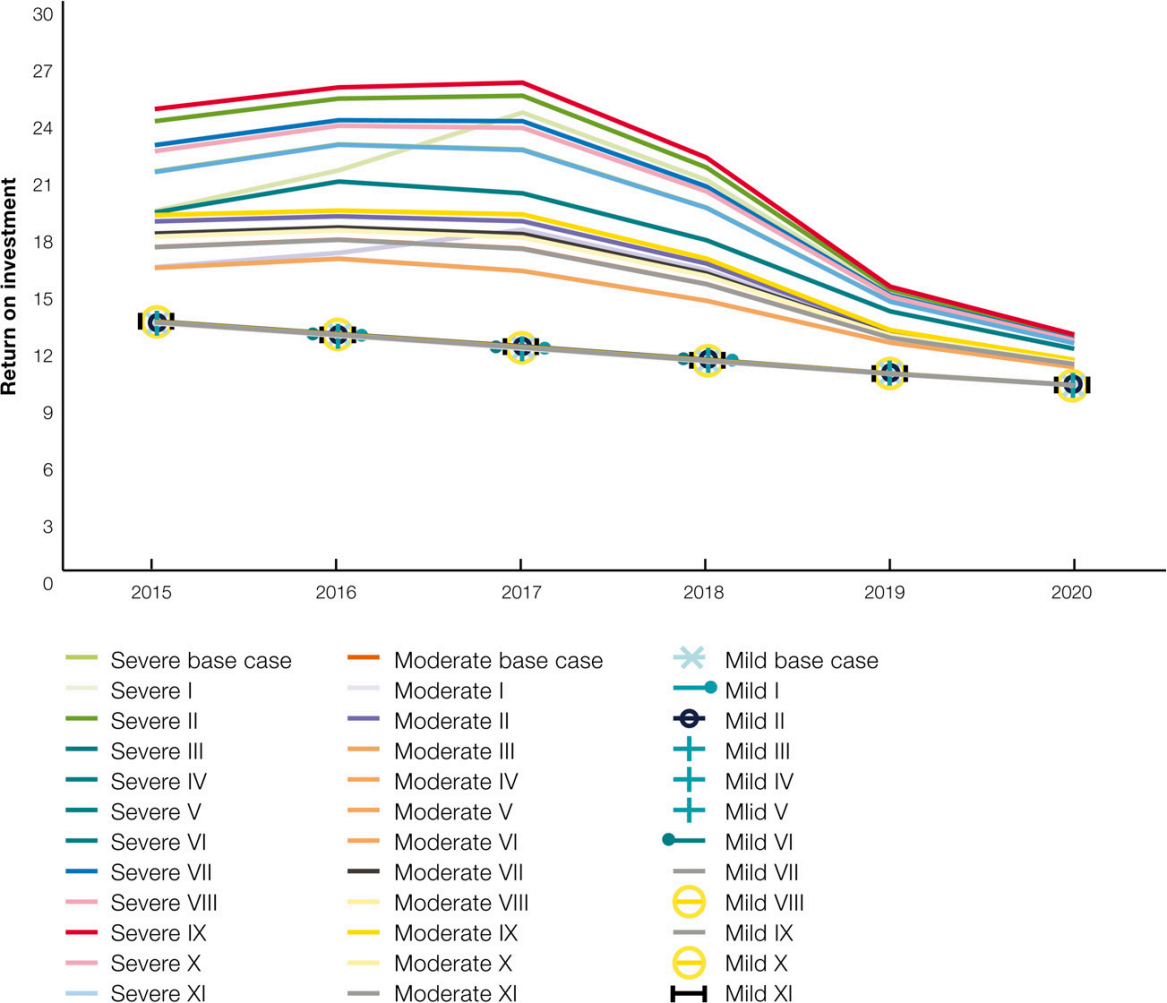
Sensitivity analysis of the estimates of return on investment in malaria using financial costs.

illustrates the sensitivity of the ROI using financial cost to varying levels of risk and probability of resurgence. Eleven scenarios of incidence were used in the sensitivity analysis denoting risk at three levels of probability; 100%, 51%, and 2% for a total 33 scenarios (Table 4).

### Financing for malaria.

In 2014, total funding for malaria activities from all sources was USD8.7 million, which accounted for about 1% of the overall government spending on health in Sri Lanka.^5^ Domestic funding accounted for about 58% of the expenditure on malaria in the country in 2014, whereas the remaining 42% of the funding for malaria came from the Global Fund at USD3.7 million.^5^ Table 7 provides the actual and the projected expenditures on malaria from 2012 to 2017.

The financial cost required to maintain the current level of malaria activities in Sri Lanka in 2015 was estimated to be on average about USD7,673,961 million annually. Domestic financing covered approximately 53% at USD4,054,878. Even with resources from the Global Fund at approximately USD2.3 million, Sri Lanka still faces a financial gap of about USD1.7 million annually.

## Discussion

This study found that the economic cost of maintaining malaria POR in Sri Lanka was approximately USD0.57 per capita in 2015 and the corresponding financial cost was USD0.37 per capita. In contrast, the cost of resurgence in 2015 was estimated to be USD169 million or USD8.07 per capita in a single year, yielding an economic ROI of 13.29 to 1 and a financial return of 21 to 1. This by far exceeds the threshold on returns that are considered to be high-impact investments.

The estimates of cost of resurgence in this study are likely to be undervalued as they exclude several macroeconomic costs of malaria far beyond the health system. Studies have shown that indirect costs of malaria account for a large share of societal costs due to its debilitating physical impact leading to cognitive disability in children and later productivity as adults, as well as impeding macroeconomic development by limiting foreign investments and tourism.^25^–^29^ These macroeconomic impacts have not been included in these estimates, primarily due to the lack of accurate data to quantify these effects and to directly attribute them to malaria. Other costs to the health system such as cost of drug and insecticide resistance, the cost of higher price alternatives, the cost associated with their implementation, and the cost of research and development have also been omitted.

There are several limitations to the data and methods used in this study. Obtaining accurate data on the cost of program operations, particularly in an integrated health system, is challenging. Several malaria program resources were shared across other public health programs. Peripheral level staff is often designated to perform other public health functions such as dengue surveillance following the decline in malaria burden leading to difficulties in attributing specific resources to malaria alone. Furthermore, activities for malaria were paid for through a combination of government and external resources. Although most provincial level staff was paid using government funds, several central AMC staff were funded through the Global Fund grants. In addition, resources for malaria control were spread across interventions and activities. Costs for malaria in this study were estimated using self-reported hours during the interview process and apportioned to the respective malaria activities. While this is a common methodology used in other studies, the authors acknowledge the potential reporting bias in the estimates. Ideally, a protracted period of time would be spent in the field to closely monitor and record the time and resources spent on each activity. However, such an approach would require a considerably more resources than those available for this work.

The perspective used for estimating the cost of POR was the public sector provider perspective as the majority of costs incurred for malaria are from the public sector with prevention and treatment provided free by the government at the time of this analysis.

The findings of this work are based on a hypothetical resurgence scenario. Although the probability and magnitude of resurgence are difficult to predict, historical evidence from Sri Lanka and other countries suggests that weakening vigilance and waning financing provide a high risk for malaria resurgence.^7^ In this study, the cost of resurgence was over 14 times the cost of POR with a healthy ROI of 13 to 1. Varying the risk and probability of resurgence consistently outweighs the cost of investing in POR.

The major cost driver in the resurgence scenario was vector control. The analysis used conservative estimates of vector control coverage of 4% for IRS and targeted LLIN coverage to populations at risk. The authors recognize that the resulting ROI is based on these assumptions; however, historical evidence from Sri Lanka, experience from other countries, and expert consultations on the intervention coverage in a potential resurgence scenario were used to inform these assumptions.

The total income approach was used to compute income losses from malaria mortality. Although this methodology provides more generous estimates of losses than other methods, given the small number of deaths in the resurgence scenario, the use of this method is not likely to have resulted in a significantly higher than expected ROI.

There are currently no global recommendations on the specific mixes of interventions needed for elimination and POR, and little data on the effectiveness and cost-effectiveness of the various strategies for POR. The AMC has largely suspended vector control activities in favor of rigorous epidemiological and entomological surveillance. Decisions on intervention selection were made by experts with in-depth historical knowledge of malaria epidemiology in Sri Lanka, bolstered by pragmatic decision-making. These cost estimates are largely founded on the assumption that the current strategy in Sri Lanka will continue to succeed in preventing POR. Nevertheless, without a transmission model or comparative trial data to assess the epidemiological and economic efficiency of the intervention mix, it is difficult to recommend optimal strategies or to judge if further cost savings can be accrued through technical and programmatic efficiencies.

When compared with projected “top-down” cost estimates from the NSP, the economic cost is approximately 43% higher as our estimates include societal costs to the health system including health worker salaries in the integrated health system. Using financial costs only demonstrated similar estimates to the NSP projections with a financial cost of 7% less than the top-town budget projections. In addition, the NSP projections do not include the savings that the AMC had accrued from insecticide procurement from targeting IRS to high-risk areas.

Despite the robust benefits associated with investing in malaria POR, Sri Lanka's program is likely to face a gap in funding in the immediate future. Funding for malaria from government sources met only 53% of the total needs in the country, as estimated by this study. This gap is likely to be much higher after 2018 when the Global Fund grant ends, which unless bridged by domestic resources will result in a severe funding cliff with potential devastating effects on the malaria program.

Despite the waning commitment from donors and shifting of government priorities, there are several opportunities within the country to mobilize additional resources for POR. Sri Lanka currently allocates only about 0.43% of their total domestic expenditure on health to malaria. A recent analysis by Jha and colleagues suggest that if Asian countries were to allocate two percent of their health budgets to malaria, the funding gap would be reduced significantly.^30^ Increasing the funding domestically or identifying alternative financing mechanisms is imperative to sustaining the gains in malaria control and elimination in Sri Lanka.

Sri Lanka's economy has experienced strong growth rates in recent years. The flourishing economy presents an opportunity for the government to increase its domestic allocations for health and hence funding for malaria. Tax revenues constitute only around 13.1% of Sri Lanka's total GDP in 2013, although the government of Sri Lanka has recently announced new adjusted tax proposals.^31^,^32^ Raising tax revenues to amount to 20% of GDP as recommended by the Addis Ababa accord for the Sustainable Development Goals would generate an additional revenue of USD4.35 million per year—a potential funding source for malaria POR.^33^ The private sector is also a major player in Sri Lanka's economy. A total of 40 companies collectively spend about USD30.5 million annually on corporate social responsibility (CSR) covering a wide range of development issues.^12^ The CSR consortia in Sri Lanka has recently partnered with Sri Lanka's Public Health Department for dengue eradication. Tapping into the resources from CSR programs of large multinational firms operating in Sri Lanka to fight malaria may also be a potential resource for POR. Sri Lanka has already adopted a policy for discouraging alcohol consumption and smoking by raising taxes on both products in recent years providing additional government revenue. Exploration of other means of augmenting domestic financing using innovative approaches such as health and diaspora bonds and airline and financial transaction taxes have the potential to supplement government revenue, which can be used for health including malaria.^21^

High-level advocacy to policy makers and donors is needed to ensure sustained financing for malaria. This study provides compelling evidence on the economic benefits of continued prioritization of funding for malaria, which can be used to strengthen the advocacy argument for increased domestic and external funding to keep Sri Lanka malaria free.

## Figures and Tables

**Table 1:** Detailed explanation of cost categories

Vector control diagnosis	Environmental management
	Targeted biological control
	Personal and community protection (LLINs and IRS)
	Chemical larviciding
	Rapid diagnostic test
	Molecular diagnosis and confirmation
	Quality assurance
Treatment and prophylaxis	Chemoprophylaxis
	Passive case detection and treatment
	Provider training
Surveillance and epidemic management	Active case detection
	Activated passive case detection
	Entomological surveillance
	Case investigation and response
	Epidemic response
	Surveillance training
	Private sector surveillance
Monitoring and evaluation (ME)	Internal ME
	External ME
	Health information system
	Periodic surveys
Information, education, and communication	Private sector engagement
	Partnership development
	Behavior change communication programs
	Policy advocacy
	School-based education
	Operational research
Program management	Administrative training
	Capacity building
	Staff placement and recruitment
	Meetings
	Supervision and monitoring
	General administration

Each of the categories above includes the human resources, consumables, and utility costs associated with implementing the activity.

**Table 2:** Input parameters and the data sources

Parameter	Values	Source	Comments
Population	18.75 million (year 1999)	12	Projected for 2015 based on population growth rates from United Nation^13^
	20.96 million (year 2015)		
GDP per capita	Year 1999: 2135.7 (in 2005$)	14	
	Year 2015: 3839		
GDP growth rate	Year 2015: 7.4%	14	
Malaria			
Number of cases	264,549 (year 1999)	AMC database (unpublished data)	Projected for 2015 based on population growth rates from UN
	324,371 (year 2017)		
Distribution of cases by gender	Male: 54% (1999); 90% (2015)	AMC database (unpublished data)	Distribution for year 2015 based on that for 2011
	Female: 46% (1999); 10% (2015)		
Distribution of cases by age	< 15 years: 41% (1999): 6% (2015)	AMC database (unpublished data)	Distribution for year 2015 based on that for 2011
	> 15 Years: 59% (1999): 94% (2015)		
Number of deaths	102 (1999)	AMC database (unpublished data)	Projected for 2015
	122.3 (2015)		
Proportion of uncomplicated cases	75%	AMC database (unpublished data)	
Proportion of severe cases	25%	AMC database (unpublished data)	
Proportion *vivax*	76%	AMC database (unpublished data)	
Proportion *falciparum*	24%	AMC database (unpublished data)	
Slide positivity rate	16.72%	AMC database (unpublished data)	
Total blood films	1.58 million	AMC database (unpublished data)	
% population protected by IRS	4% twice a year	AMC database (unpublished data)	
No. of LLINs needed	1 LLIN per 1.8 population in “at risk areas”	15	
Cost and related parameters			
No. of days lost due to a malaria illness	9.3 days	16	
Cost of OP illness	USD1.68	AMC database (unpublished data)	
Cost of IP admittance	USD24.49	AMC database (unpublished data)	
Cost of malaria medicines (OP)	USD1.00	AMC database (unpublished data)	
Cost of malaria medicines (IP)	USD8.5	AMC database (unpublished data)	
Cost of IRS per person protected	USD4.37	18	
Cost of LLIN distributed	USD6.87	18	
Cost of testing non-malaria fevers	USD1.12 per RDT	18	
	USD0.86 per microscopy slide		
Cost for sulfadoxine pyrimethamine during pregnancy	USD0.5	Kurunegala Teaching Hospital (unpublished data)	
Cost of household consumption goods for malaria	USD7.31	16	
Tourism			
Number of tourists (in million)	0.44 million (1999)	19	
	1.89 million (2015)		
Average nights spent by tourist	8.6 (1999)	19	2015 data is based on author's projection based on previous trends
	9.25 (2015)		
Average revenue per tourist per day	USD158.65	19	
Percentage of tourists from Europe and North America	67	19	

LLIN = long-lasting insecticidal net; RDT = rapid diagnostic tests.

**Table 3:** Treatment guidelines for malaria treatment in Sri Lanka

Uncomplicated malaria (*Plasmodium falciparum*)	Hospitalization for 3 days with immediate dose of primaquine (0.75 mg/kg body weight) plus artemether–lumefantrine (20/120 mg)
Severe malaria (*P. falciparum*)	Hospitalization with injectable artesunate until patient can take medication orally (usually 3 days) after which a complete course of artemether–lumefantrine (20/120 mg) is given
Military	*Plasmodium vivax* patients hospitalized for 3 days in military medical facilities; patients are kept within their barracks for 2 weeks for 14-day primaquine regimen (0.25 mg/kg body weight) in addition to chloroquine for 3 days
Non-military	Primaquine for 14 days (0.25 mg/kg body weight) plus chloroquine for 3 days
Mixed infections	Artemether–lumefantrine (20/120 mg) for 3 days plus primaquine for 14 days as an inpatient for 3 days

**Table 4:** Scenarios for uncertainty analysis

Severity of resurgence	Scenarios
Incidence rate similar to historical rates between 1997 and 2002	Baseline
Maximum annual growth rate observed between 2 peak years	I
Maximum total growth rate observed between 2 peak years	II
Growth rate in 1975 from previous trough year	III
Growth rate in 1987 from previous trough year	IV
Growth rate in 1991 from previous trough year	V
Growth rate in 1999 from previous trough year	VI
Growth rate required to reach number of cases in year 1968 from 2012 level	VII
Growth rate required to reach number of cases in year 1975 from 2012 level	VIII
Growth rate required to reach number of cases in year 1987 from 2012 level	IX
Growth rate required to reach number of cases in year 1991 from 2012 level	X
Growth rate required to reach number of cases in year 1999 from 2012 level	XI
Probability of resurgence	
100%	Severe
51%	Median
2%	Mild

The severity of resurgence is determined based on a combination of historical growth rates since 1950 to reach the peak level of resurgence from the base year 2012 (when only 23 cases were observed). The distribution of cases during hypothetical resurgence years (2015–2010) followed the actual case distribution observed between years 1997–2002.

**Table 5:** Projected cost for malaria prevention of reintroduction

Year	Estimated annual cost (millions USD)	Cumulative cost (millions USD)
2015	11.86	11.86
2016	12.62	24.48
2017	13.43	37.90
2018	14.28	52.19
2019	15.20	67.39
2020	16.17	83.56

**Table 6:** Cost of resurgence of malaria for year 2015

Cost of resurgence in 2015	Best estimate (in millions USD)
Direct cost to the health system	
Cost due to increased health service utilization	14.63
Cost of vector control to control resurgence	104.08
Cost of increased diagnosis	1.30
Cost of training human resources and educating community	1.31
Direct cost to the individual household	
Out of pocket expenditure due to malaria	1.96
Indirect cost to the society	
Cost due to loss of life to malaria	21.13
Cost due to loss of productivity to malaria morbidity	24.54
Total cost of resurgence in 2015	**168.96**

**Table 7:** Actual and projected expenditures for the malaria program in Sri Lanka 2012–2017

Source of funding	Actual funds spent (millions USD)			Projected funds (USD)		
	2012	2013	2014	2015	2016	2017
Domestic spending*	3.26	3.63	5.06	5.49	6.12	6.77
Global Fund support†	2.91	3,.13	3.72	2.47	2.47	2.47
Total budget for malaria control	6.17	6.76	8.78	7.95	8.58	9.23
Total domestic spending on health	7.58	8.41	9.34	1,037	1,151	1,277
% of domestic funding for malaria	53	54	58	69	71	73
% of domestic health budget allocated for malaria	0.43	0.43	0.54	0.53	0.53	0.53
Total budget for malaria as a percentage of total domestic spending on health	0.81	0.80	0.94	0.77	0.75	0.72

* Based on data published by the Central Bank of Sri Lanka (www.cbsl.gov.lk).

† Global fund support amounting to USD9.6 million has been requested for the period 2014–2017. Given that this grant was not approved until 2015, it has been allocated to 2015–2017 projected costs and has been split evenly among the 3 years.
